# Supplementary material for: MetaRibo-Seq measures translation in microbiomes
Source: Nat Commun. 2020 Jun 29;11:3268. doi: 10.1038/s41467-020-17081-z (PMC7324362; doi:10.1038/s41467-020-17081-z)
Supplement: Supplementary file 10 — Supplementary Data 7 [file 41467_2020_17081_MOESM10_ESM.zip › File2/Confidence_High_Taxonomy/261667_out.krona.html]

Javascript must be enabled to view this page.

members
magnitude
magnitudeUnassigned
count
unassigned
taxon
rank

261667\_out

3

2
10239
superkingdom

2
family
10841

species
2202644

SRS140492\_contig\_number\_7326
1

1
686565
subfamily

species
1758150
1

SRS023971\_contig\_number\_contig-100\_58.58

1
2
superkingdom

1
phylum
204428

1
204429
class

1
order
51291

1
family
809

genus
810
1

813
species

SRS013965\_contig\_number\_20516
1
